# Optic nerve injury in preoperative imaging is associated with visual improvement outcome in endoscopic optic nerve decompression

**DOI:** 10.1007/s00508-021-01915-x

**Published:** 2021-08-03

**Authors:** Isabella Leitner, Alexandros Andrianakis, Verena Gellner, Peter Kiss, Damianos Andrianakis, Peter Valentin Tomazic

**Affiliations:** 1 Department of Otorhinolaryngology, Head and Neck Surgery, Medical University of Graz, Auenbruggerplatz 26, 8036 Graz, Austria; 2 Department for Neurosurgery, Medical University of Graz, Graz, Austria; 3 Institute of Mathematics and Scientific Computing, University of Graz, Graz, Austria

**Keywords:** Acute optic neuropathy, Optic nerve sheath incision, Steroid treatment, Endoscopic sinus surgery

## Abstract

**Objective:**

To evaluate potential clinical parameters having an impact on visual outcome after endoscopic optic nerve decompression in acute optic neuropathy patients.

**Methods:**

A retrospective chart review of patients with acute optic neuropathy, who underwent endoscopic optic nerve decompression between June 2001 and November 2018 at an academic center was performed. Patients were divided into groups according to visual improvement after surgical treatment (yes/no). Following clinical parameters were compared between groups: perioperative steroid use, evidence of optic nerve affection in preoperative neuroimaging, additional optic nerve sheath incision, surgery delay and preoperative C-reactive protein (CRP) levels. Further subgroups analyses were conducted based on etiology (trauma/tumor).

**Results:**

Among 32 included cases, 16 patients (50%) reported visual improvement after endoscopic optic nerve decompression. There was no significant difference in visual improvement between etiology subgroups (trauma: *n* = 9/20 (45%) vs. tumor: *n* = 7/12 (58.3%), *p* = 0.465). Tumor subgroup patients with visual improvement had a significantly higher prevalence of optic nerve affection in preoperative neuroimaging than those without visual improvement (*p* = 0.018, φ = 0.683). Perioperative steroid administration was negatively associated with visual outcome (*p* = 0.034, φ = 0.375). Nerve sheath incision, surgery delay and preoperative CRP levels did not have a significant impact on visual outcome (*p* > 0.05).

**Conclusion:**

Radiological findings can help as an indicator for surgical treatment since an affected optic nerve in preoperative neuroimaging resulted in better visual outcome after surgery. The use of steroids should be considered more carefully since it did not show any beneficial effect.

## Introduction

Acute optic neuropathy can be defined as acute damage to the optic nerve resulting in prompt ophthalmologic symptoms like blurry vision, decreased color vision, scotomas, visual field defects and visual impairment. Causes include optic nerve ischemia, inflammation, tumor compression and traumatic optic nerve injury [1].

The diagnosis is based on medical history, physical examination and neuroimaging. Survey of patient’s anamnesis should involve besides the general medical history (e.g. pre-existing comorbidities, medication intake, family medical history) and complaints/symptoms, the presence of a recent trauma in order to quickly differentiate between a traumatic or nontraumatic etiology. The physical examination is focused on the ophthalmological assessment, including the measurement of the visual acuity, pupillary light reaction, fundoscopy and perimetry. Neuroimaging is obligatory for evaluation of optic nerve lesions and signs of fracture. Computed tomography (CT) is often the preferred primary imaging modality, especially in traumatic cases [1–5]. The examination is fast, cost-effective, not limited by the use of potential foreign bodies and it excellently detects bony fractures and bony fragments comprising the optic nerve. Moreover, CT imaging can identify lesions nearby the optic nerve and inside the orbit, causing damage to the nerve by direct compression or infiltration [6–8]. Nevertheless, magnetic resonance imaging (MRI) is the gold standard in the diagnosis of soft tissue. Therefore, particularly in nontraumatic cases the MRI is the imaging modality of choice for a precise evaluation of the orbit, optic nerve and surrounding area [9–11].

The therapeutic management primarily targets the improvement and maintenance of visual function. In this context, the optimal therapeutic approach in literature appears inconstant. Currently available treatment options are corticosteroids and surgical optic nerve decompression [12, 13].

The therapeutic use of corticosteroids for acute optic neuropathies is mainly based upon their ability to relieve inflammations and edema, being neuroprotective and antioxidative [14–16]. Possible positive effects of steroids are suppression of immunological mediators, shorter hospitalization and a better recovery time [17]. An experimental study of Lew et al. [18] showed that a high-dose steroid therapy can increase the blood flow of the optic nerve in rabbits. Visual improvement was seen in patients who were treated within a short time frame after injury [14–16]. Reported harmful effects, especially due to high doses, are increased mortality, a worse survival rate and increased axonal loss [16, 19, 20]. A clear benefit of steroids and when they should be given has not been stated clearly thus far [6, 21]. Moreover, they may be given in combination with surgery or as a single medical treatment [14–16].

Along with the introduction of functional endoscopic sinus surgery and the subsequently developed endoscopic skull base surgery, surgical decompression of the optic nerve as treatment option for traumatic optic nerve injuries and compressive optic neuropathies gained popularity in the past decades. Indications for surgical optic nerve decompression include a traumatic nerve injury and compressive neuropathies, e.g. adenomas of the pituitary gland (incidence 74–94/100,000 cases per year), fibrous dysplasia (incidence 15/100,000 cases per year) endocrine orbitopathy (incidence 2.9–16/100,000 cases per year), aneurysm close by the optic nerve (incidence 1.4/100,000 cases per year), craniopharyngiomas (incidence 0.2–5/100,000 cases per year) and optic nerve sheath meningiomas (2% of orbital tumors, very rare and associated with neurofibromatosis type 2). In general, incidence of compressive optic neuropathy is approximately 4/100,000 cases per year [3, 10, 22–26]. Endoscopic optic nerve decompression should be performed when there is a decrease in visual acuity after an injury or compression and the optic nerve is still intact [3]. This surgery allows to avoid further damage to the nerve which is caused by edema. Moreover, it enables removal of potential bone fragments that may compromise the nerve [27]. Surgery should not be performed if the optic nerve is completely avulsed (because the nerve will not recover), the shape of the bulb has changed distinctly, anatomical difficulties do not permit surgery or if the patient is not amenable for surgery in general. The transnasal endoscopic approach for optic nerve decompression, performed by otorhinolaryngologists, is considered as gold standard providing many benefits including reduced mortality, faster recovery of patients and the minimally invasive approach (which results in a better cosmetic outcome). With the endoscopic approach, the medial, inferior and superior parts of the optic nerve canal can be reached through the lateral wall of the sphenoid sinus. If the lateral part is mainly affected, a craniotomy approach, carried out by a neurosurgeon, may be indicated [2, 3, 28].

During surgery an incision of the optic nerve sheath can be performed for additional decompression. The benefit of this procedure is still not clear. Optic nerve sheath incision can reduce the risk for a compartmental syndrome after an injury but also carries risks such as cerebrospinal fluid leakage [3, 9, 29–31].

Regarding the optimal timing of surgery, there are different opinions on this subject matter. Yang et al. [32] achieved in 96 patients suffering from traumatic optic neuropathy the best results within a time frame of more than 3 days between injury and surgery. Tandon et al. [5] recommend 1–2 weeks as an interval. In an interventional study with 133 patients, Levin et al. [33] could not find a benefit from treating an optic nerve lesion immediately after injury. In their prospective case study of 20 patients, Gupta et al. [34] observed the best results within a time frame of 72 h to perform surgery. Dhaliwal et al. [12] summed up the controversial findings in a systematic review of 24 articles regarding the timing of intervention. According to them, no specific point of time is favored, but even late interventions may help to improve vision.

At our otorhinolaryngology department, the endoscopic optic nerve decompression as treatment modality for optic nerve injury has been performed since approximately two decades; however, there are no existing guidelines on surgical treatment of optic nerve injuries. This might be due to the paucity of clinical studies regarding this treatment option. Hence, the precise therapeutic benefit of surgical optic nerve decompression remains unknown. Moreover, there is no consensus on optimal timing of surgery and the beneficial effect of additional optic nerve sheath incision as well as perioperative corticosteroid use [12, 13].

Taking these facts into account, we retrospectively evaluated in the present study all patients, who underwent endoscopic optic nerve decompression at our department, and analyzed clinical parameters, which may have had an impact on visual outcome in order to adapt the treatment management for future cases and to help establish guidelines for this treatment modality.

## Material and methods

### Study design

In this retrospective cohort study, data from patients at a tertiary referral center, who underwent a surgical optic nerve decompression between June 2001 and November 2018, were collected. Patients with recent surgery at the optic nerve area, a decompression of the orbit, and craniotomized patients were excluded. Collected data were statistically analyzed to compare clinical parameters that may have favored visual improvement after surgery. Therefore, patients were divided into groups according to visual improvement after surgical optic nerve decompression (yes vs. no). Visual improvement was determined by patient’s subjective self-reports. Further subgroup analyses were conducted based on etiology (trauma vs. tumor). Assessed relevant parameters were: use of steroids (yes/no), radiological evidence of an affected optic nerve (yes/no), nerve sheath incision (yes/no), elevated C‑reactive protein (CRP) levels before surgery (CRP < 1 mg/l, 1 < x < 10 mg/l, >10 mg/l) and time frame between injury/visual deterioration and surgery (<1 day/1–3 days/>3 days/>1 week).

### Statistical analysis

SPSS © statistical software, version 25.0 (IBM ©, Armonk, NY, USA) was used for statistical analysis. Statistical significance level was set at *p* < 0.05, two-sided. Continuous variables are presented as means ± standard deviations in case of normal distribution and as median together with range in absence of normal distribution. Normal distribution was assessed by Kolmogorov–Smirnov test. Categorical variables are expressed as absolute numbers and percentages. For comparison of categorical variables, χ^2^-test was utilized. In cases of expected cell frequencies less than 5, the exact test method was used. The effect size of statistically significance differences in Chi-squared tests was expressed by φ‑coefficient.

### Ethical considerations

This study was independently reviewed and approved by the institutional local ethics committee (approval number: 30-216 ex 17/18) and was performed in accordance with the ethical guidelines of the Declaration of Helsinki. Due to the retrospective nature of this study, patient’s informed consent was not obtained because clinical records were anonymized prior to analysis.

## Results

**Fig. 1:**
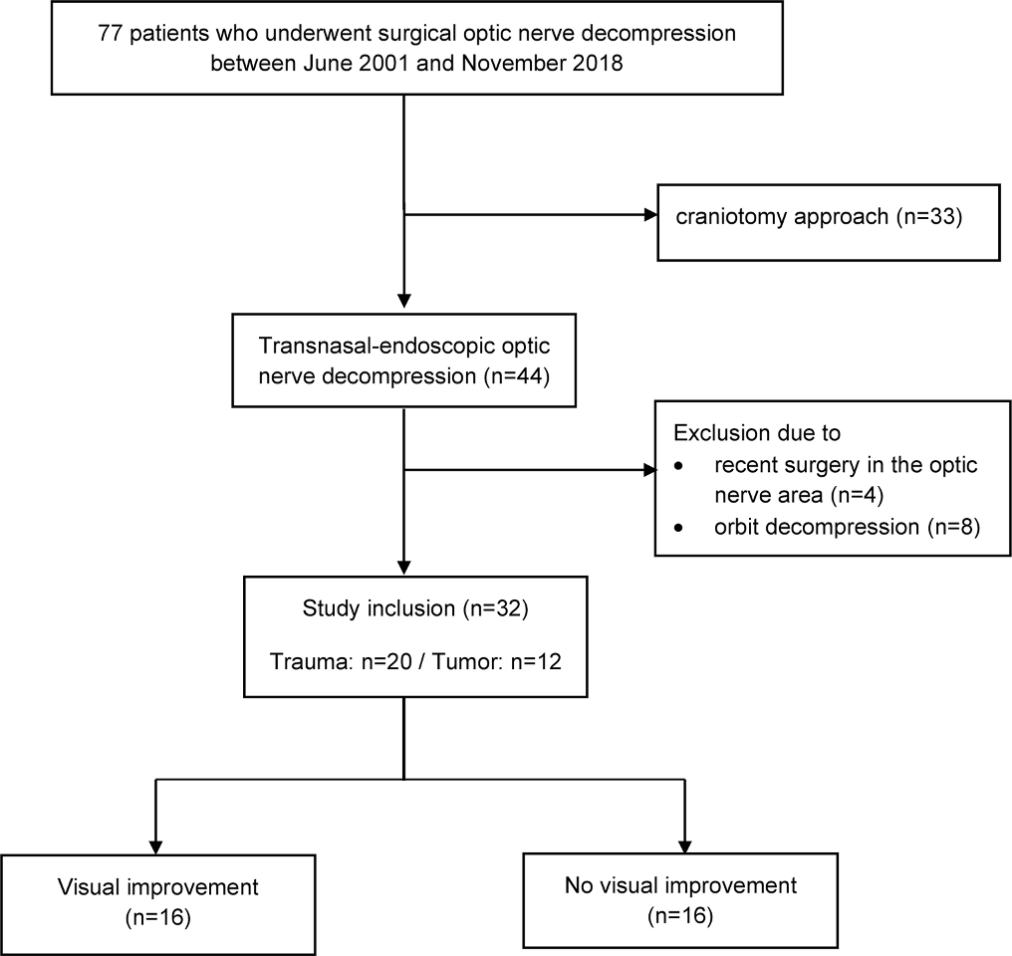
Study flow diagram

Between June 2001 and November 2018, 77 patients underwent surgical optic nerve decompression at our academic center. In 33 cases, a craniotomy was used as surgical approach. These patients were treated at the institution’s Department of Neurosurgery and, therefore, precise data were not available for additional analysis and direct outcome comparison. Further 12 patients were excluded: reasons for exclusion are displayed in the study flow diagram (Fig. 1). At the total end, we analyzed 32 eligible patients who were treated with endoscopic optic nerve decompression at our institution’s Department of Otorhinolaryngology.

Our cohort included 12 females (37.5%) and 20 males (62.5%) with an average age of 45.3 years (SD: 19.3 years). Patient’s demographic data are summarized in Table 1. Visual improvement was achieved in 16 patients (50%). Optic nerve affection was caused by traumatic injury in 20 cases (62.5%) and by a tumor in 12 cases (37.5%). There was no significant association between etiology and visual improvement outcome (visual improvement/trauma: *n* = 9/20 (45%), visual improvement/tumor: *n* = 7/12 (58.3%); χ^2^(1) = 0.533, *p* = 0.465). Traffic accidents or accidents at work (e.g. construction sites) are reported in the medical records as main traumatic injury cause. Regarding the tumor entities, the most frequent were fibrous dysplasia and meningioma with an incidence of 25% (*n* = 3) each.

**Table 1:** Patient’s demographic data

	Total cohort	Vision improvement			No vision improvement			*p*-value
	*n* = 32	Total* n = 16	Trauma** n = 9	Tumor*** n = 7	Total* n = 16	Trauma** n = 11	Tumor*** n = 5	
*Age, years*	45.3 (SD 19.3)	41.7 (SD 16.5)	36.2 (SD 17.4)	48.8 (SD 13.4)	48.9 (SD 21.6)	47.1 (SD 21.1)	52.8 (SD 24.9)	*t(30) = 1.1, *p* = 0.301 **t(18) = 1.2, *p* = 0.229 ***t(10) = 0.3, *p* = 0.729
*Sex, n (%)*								*χ^2^(1) = 0.5, *p* = 0.465 **χ^2^(1) = 0.1, *p* = 0.769 ***χ^2^(1) = 0.3, *p* = 0.558
Female	20 (62.5)	7 (43.8)	3 (33.3)	4 (57.1)	5 (31.3)	3 (27.3)	2 (40)	–
Male	12 (37.5)	9 (56.3)	6 (66.7)	3 (42.9)	11 (68.2)	8 (72.7)	3 (60)	–

*SD* standard deviation

During optic nerve decompression, a nerve sheath incision was performed in 15 patients (48.4%). In one case, surgical report was not sufficiently documented. The remaining 16 patients (51.6%) did not have an incision. There was no statistically significant difference in nerve sheath incision rate between visual improvement groups (χ^2^(1) = 0.82, *p* = 0.366). Detailed results are displayed in Table 2. All of the optic nerve sheath incisions were performed in traumatic injury cases (*n* = 15/20, 75%).

**Table 2:** Impact of optic nerve sheath incision during surgery on visual improvement

	Total (*n* = 31)	Vision improvement (*n* = 15)	No vision improvement (*n* = 16)
Nerve sheath incision, *n* (%)	15 (48.4)	6 (40)	9 (60)
No nerve sheath incision, *n* (%)	16 (51.6)	9 (56.3)	7 (43.7)

χ^2^(1) = 0.82, *p* = 0.366

The time frame between injury/vision deterioration and surgery ranged between less than 1 day and more than 6 months. To test a statistical relationship, time frame was divided into four categories: <1 day (*n* = 8), 1–3 days (*n* = 3), >3 days (*n* = 7), >1 week (*n* = 14). There were no statistically significant differences in prevalence of time categories between visual improvement groups, neither in the total cohort analysis (χ^2^(3) = 2.12, *p* = 0.614), nor in the trauma subgroup (χ^2^(3) = 2.94, *p* = 0.580) and tumor subgroup (χ^2^(3) = 0.89, *p* = 0.827). Detailed results are depicted in Table 3.

**Table 3:** Impact of time frame between injury/vision deterioration and surgery on visual improvement

	Total cohort	Vision improvement			No vision improvement		
	*n* = 32	Total* n = 16	Trauma** n = 9	Tumor*** n = 7	Total* n = 16	Trauma** n = 11	Tumor*** n = 5
<1 day	8 (25)	3 (37.5)	2 (22.2)	1 (14.3)	5 (62.5)	4 (36.4)	1 (20)
1–3 days	3 (9.4)	1 (33.3)	0 (0)	1 (14.3)	2 (66.7)	2 (18.2)	0 (0)
>3 days	7 (21.9)	3 (42.9)	2 (22.2)	2 (28.6)	4 (57.1)	1 (9.1)	2 (40)
>1 week	14 (43.8)	9 (64.3)	5 (55.6)	3 (42.9)	5 (35.7)	4 (36.4)	2 (40)

Categorical variables are presented as absolute numbers and percentages (%)

*χ^2^(3) = 2.12, *p* = 0.614

**χ^2^(3) = 2.94, *p* = 0.580

***χ^2^(3) = 0.89, *p* = 0.827

CRP levels were measured in 23 patients before surgery. The counts ranged between 0.1 mg/l and 143.9 mg/l with a median of 1.1 mg/l. CRP levels were divided into three categories: 10 patients (43.5%) had a value of <1 mg/l, 7 patients (30.4%) of 1–10 mg/l and 6 patients (26.1%) of >10 mg/l. No statistically significant differences were found in CRP categories between visual improvement groups, neither for the total cohort (χ^2^(1) = 1.64, *p* = 0.500), nor for etiology subgroups (*p* > 0.05). Detailed results are shown in Table 4.

**Table 4:** Impact of pre-op CRP levels on visual improvement

	Total cohort	Vision improvement			No vision improvement		
	*n* = 23	Total* n = 12	Trauma** n = 5	Tumor*** n = 7	Total* n = 11	Trauma** n = 6	Tumor*** n = 5
<1 mg/l CRP	10 (43.5)	4 (33.3)	2 (40)	2 (28.6)	6 (54.5)	3 (50)	3 (60)
1–10 mg/l CRP	7 (30.4)	5 (41.7)	1 (20)	4 (57.1)	2 (18.2)	1 (16.7)	1 (20)
>10 mg/l CRP	6 26.1	3 (25)	2 (40)	1 (14.3)	3 (27.3)	2 (33.3)	1 (20)

Categorical variables are presented as absolute numbers and percentages (%)

*χ^2^(2) = 1.6, *p* = 0.500

**χ^2^(2) = 1.1, *p* = 0.946

***χ^2^(2) = 1.7, *p* = 0.747

Perioperative steroids were administered highly-dosed (250 mg prednisolone) by the intravenous route. In 9 of 32 cases (28.1%), steroids were given before surgery. There were no statistically significant differences in preoperative steroid usage between visual improvement groups, neither in the total cohort analysis (χ^2^(1) = 3.8, *p* = 0.113), nor in the trauma subgroup (χ^2^(1) = 2.8, *p* = 0.157) and tumor subgroup (χ^2^(1) = 1.1, *p* = 0.523). Detailed results are displayed in Table 5. In 16 cases (50%), patients received steroids before and after surgery. There was no case in which a patient received solely postoperative steroids. Administration prevalence of steroids (preoperative or preoperativeop + postoperative) differed statistically significant between visual improvement groups (χ^2^(1) = 4.5, *p* = 0.033, φ = 0.378). Patients without any steroid treatment had a 37.5% significantly higher prevalence of visual improvement in comparison to patients who received steroids. Concerning etiology subgroup analysis (trauma vs tumor), there were no statistically significant differences in steroid usage between visual improvement groups (*p* > 0.05). Exact details are presented in Table 6.

**Table 5:** Impact of pre-operative steroids on vision improvement

	Total cohort	Vision improvement			No vision improvement		
	*n* = 32	Total* n = 16	Trauma** n = 9	Tumor*** n = 7	Total* n = 16	Trauma** n = 11	Tumor*** n = 5
Preoperative steroids	9 (28.1)	2 (12.5)	1 (11.1)	1 (14.3)	7 (43.8)	5 (45.5)	2 (40)
No preoperative steroids	23 (71.9)	14 (87.5)	8 (88.9)	6 (85.7)	9 (56.2)	6 (54.5)	3 (60)

Categorical variables are presented as absolute numbers and percentages (%)

*χ^2^(1) = 3.8, *p* = 0.113

**χ^2^(1) = 2.8, *p* = 0.157

***χ^2^(1) = 1.1, *p* = 0.523

**Table 6:** Impact of steroid use (preoperative or preoperative + postoperative) on visual improvement

	Total cohort	Vision improvement			No vision improvement		
	*n* = 32	Total* n = 16	Trauma** n = 9	Tumor*** n = 7	Total* n = 16	Trauma** n = 11	Tumor*** n = 5
Steroids were used	16 (50)	5 (31.3)	4 (44.4)	1 (14.3)	11 (68.8)	8 (72.7)	3 (60)
No steroids were used	23 (50)	11 (68.8)	5 (55.6)	6 (85.7)	9 (31.3)	3 (27.3)	2 (40)

Categorical variables are presented as absolute numbers and percentages (%)

*χ^2^(1) = 4.5, *p* = 0.034

**χ^2^(1) = 1.6, *p* = 0.199

***χ^2^(1) = 2.7, *p* = 0.098

From 32 patients, 18 (56.2%) showed an affected optic nerve (compression, edema/hematoma, tumor contact, thickening, fracture of the optic channel/foramen, gas accumulation around the nerve) in preoperative radiological imaging. There was a statistically significant difference in radiological nerve affection prevalence between visual improvement groups in the total cohort (χ^2^(1) = 4.57 *p* = 0.033, φ = 0.378). According to further conducted etiology subgroup analyses, a significant difference in preoperative radiologic evidence of optic nerve affection between visual improvement groups was solely found in the tumor subgroup (χ^2^(1) = 5.6, *p* = 0.018, φ = 0.683), not in the trauma subgroup (χ^2^(1) = 0.7, *p* = 0.391). Remaining details are depicted in Table 7.

**Table 7:** Impact of radiological evidence of optic nerve affection on visual improvement

	Total cohort	Vision improvement			No vision improvement		
	*n* = 32	Total* n = 16	Trauma** n = 9	Tumor*** n = 7	Total* n = 16	Trauma** n = 11	Tumor*** n = 5
Radiological evidence	18 (56.3)	12 (75)	5 (55.6)	7 (100)	6 (37.5)	4 (36.4)	2 (40)
No radiological evidence	14 (43.7)	4 (25)	4 (44.4)	0	10 (56.3)	7 (63.6)	3 (60)

Categorical variables are presented as absolute numbers and percentages (%)

*χ^2^(1) = 4.57, *p* = 0.033, φ = 0.378

**χ^2^(1) = 0.7, *p* = 0.391

***χ^2^(1) = 5.6, *p* = 0.018, φ = 0.683

## Discussion

In the present study we retrospectively evaluated 32 patients with acute optic neuropathy who were treated with transnasal endoscopic optic nerve decompression between June 2001 and November 2018 at our academic center. We could identify two clinical parameters which were significantly associated with the visual outcome after the surgical procedure: the radiological evidence of an affected/injured optic nerve in preoperative neuroimaging and the perioperative usage of corticosteroids.

Out of the 32 included patients, 50% (*n* = 16) reported a visual improvement following endoscopic optic nerve decompression. The optic neuropathy was caused by traumatic injury in 20 cases (62.5%) and by a tumor compression in 12 cases (37.5%). We found no significant association between etiology and visual improvement outcome: Trauma patients showed a visual improvement rate of 45% (*n* = 9/20) while a visual improvement occurred in 58% (*n* = 7/12) of the tumor patients.

In the total cohort, 18 patients had radiological evidence of an affected/injured optic nerve in the preoperative imaging. Out of these, 12 patients improved in vision after surgery. We observed a significantly better visual outcome if radiological evidence of damage to the optic nerve was present: according to our statistical analysis, there was a 38% significantly higher rate of visual improvement when the optic nerve was affected/injured in preoperative imaging (*p* = 0.033, φ = 0.378); however, it is important to further separate traumatic and nontraumatic cases in this context, due to their underlying etiology and received imaging modality. All patients in our cohort initially underwent CT imaging. CT is often the preferred primary imaging modality as it is fast, cost-effective, not limited by potential foreign bodies, can identify lesions nearby the optic nerve and inside the orbit, and it excellently detects bony fractures and bony fragments comprising the optic nerve [6–8]. Moreover, CT is advantageous for the preoperative evaluation of the individual sinonasal anatomy, when planning a transnasal endoscopic optic nerve decompression. In our cohort, 20 patients had a traumatic optic neuropathy with an improvement in visual function after surgery in 9 cases. These cases with visual improvement showed in 56% an optic nerve affection in the preoperative CT imaging, while those patients without visual improvement had an optic nerve injury in preoperative imaging in 44%. Our results correlate with findings from previous studies: Gupta et al. [34] evaluated 20 patients with traumatic optic neuropathy and optic nerve affection in the CT neuroimaging, who were treated with endoscopic optic nerve decompression. The authors reported a visual improvement of 55% (*n* = 11/20). Han [35] recommends in his case reports to perform an early intervention, if any bone fragments or fractures are close to the optic nerve. In contrast, Levin et al. [33] mentioned in their study that optic canal fractures and resulting damages of the optic nerve might decrease the chance of recovery. Also, they could not observe that radiological evidence in a CT scan was in a relationship with visual outcome (under consideration that they did not have a higher quantity of CT findings). Clearly, CT is the optimal imaging modality in acute optic neuropathies with a traumatic etiology; however, in nontraumatic cases, MRI is definitely the preferable neuroimaging modality, as it provides precise anatomic details for the optic nerve tissue itself [37]. In the present study, 12 patients had a nontraumatic, tumorous etiology and 7 of these cases (58%) improved in visual function after the endoscopic optic nerve decompression. All nontraumatic cases received an additional MRI before the surgical treatment. We observed a significant difference in optic nerve affection prevalence in preoperative neuroimaging between tumor visual improvement groups (*p* = 0.018, φ = 0.683). Out of the 9 tumor patients with optic nerve affection in preoperative neuroimaging, 7 subjects (77%) showed a visual improvement after the surgical procedure. In other words, each of the tumor patients with visual improvement (*n* = 7/7, 100%) showed evidence of optic nerve affection in the preoperative MRI. These results indicate an association between optic nerve affection in preoperative neuroimaging and visual outcome. Certainly, CT imaging is obligatory in traumatic optic neuropathy events. In nontraumatic cases where MRI is the preferred modality, the imaging result could be helpful for decision making of endoscopic optic nerve decompression. The presence of an affected optic nerve in the MRI may be used as indication for the surgical treatment as the vast majority of surgically treated cases with evidence of nerve affection in preoperative imaging showed a visual improvement.

Corticosteroids are known to improve or avoid edema, swellings, vasospasm and to regain function when treating acute optic neuropathy [34, 38]. In recent literature, several studies could not show a beneficial effect in using corticosteroids for treatment of optic nerve compression. Entezari et al. [39] performed a double-masked randomized-controlled trial in 31 patients who suffered from an indirect traumatic optic neuropathy, 16 received a steroid therapy, 15 a placebo. They could not show a significant difference in visual improvement between these groups (*p* = 0.38). Yu-Wai-Man and Griffiths [15] concluded in their systematic review that steroids do not act beneficially in treatment. According to them, the possible disadvantages of those medications should be considered. In a retrospective review of Ropposch et al. [21], no beneficial effect of steroids could be found when they were given in addition to surgery. The findings of these studies are consistent with results of the present study: 16 of the 32 included patients (50%) received some form of perioperative steroids, either solely preoperatively (*n* = 9/16) or preoperatively and postoperatively (*n* = 7/16). Our statistical analysis revealed a 37.5% significantly lower prevalence in visual improvement when steroids were administered. If the usage of steroids were causatively related with a poorer visual improvement remains certainly debatable; however, our data clearly support the opinion that steroids do not have a significant beneficial effect on visual recovery. Similar applies for the different etiologies, as we failed to find in both trauma and tumor subgroups, a benefit of steroid usage on visual outcome. Considering the lack of evidence for a beneficial effect and the potential disadvantages of corticosteroids, their usage as treatment option in traumatic and compressive optic neuropathy cases should be carefully considered.

An incision of the optic nerve sheath during the surgical treatment can be performed for an additional decompression. The precise benefit of this procedure remains unknown. Controversial results can be found in literature: Xu et al. [29] analyzed 74 patients with traumatic optic neuropathy and had a better visual outcome (65.1%) in the group without nerve sheath incision than in the group with incision (61.2%), (*p* > 0.05). This study claims that nerve sheath incision is not implicitly necessary for optic nerve decompression. In the group of patients who had a residual visual acuity before surgery, they also observed a better outcome in patients without nerve sheath incision (64.2 and 74.1%, *p* > 0.05). In contrast, Thaker et al. [9] found a beneficial effect of additional optic nerve sheath incision: in their prospective study including 57 patients with traumatic optic neuropathy treated with endoscopic optic nerve decompression, the improvement rate was higher when nerve sheath incision was performed additionally (improvement rate 46.6% with incision vs. 32.8% without, *p* = 0.10). In the present study, we failed to find a beneficial effect of optic nerve sheath incision on the visual outcome (*p* = 0.366). Nevertheless, it is essential to additionally consider the underlying cause of the optic nerve injury. Therefore, we further intended to analyze the influence of optic nerve sheath incision on the visual outcome for the different etiologies. We observed that each optic nerve sheath fenestration was performed in traumatic cases, presumably in order to decrease a trauma-induced edema. Nevertheless, there was no significant association between optic nerve sheath incision and visual improvement (*p* = 0.719). Our data indicate that additional optic nerve sheath incision does not improve postoperative visual function in traumatic optic neuropathy events. These findings are concordant with those of Xu et al. [29]. In addition to the lack of benefit of optic nerve sheath fenestration, its potential adverse events including vessel injury, CSF leak and further iatrogenic nerve damage, must be considered [36]. Unfortunately, no optic nerve sheath fenestration was performed in the tumor subgroup, hence, no conclusion can be made at this point for this kind of patients. Therefore, it remains uncertain if optic nerve sheath incision has a positive effect on the visual outcome in tumor patients, and especially in which tumor entities. For instance, patients with meningioma involving the dural optic sheath may probably benefit more from an optic nerve sheath fenestration than patients with an osseous disorder like fibrous dysplasia. However, this lack of knowledge must be addressed in a future trial.

Regarding the optimal timing of surgical treatment, there are many different opinions in literature. Several studies suggested performing surgery within 3 days to get the most benefit. By keeping to this time frame, fewer long-term damages of the nerve are reported [12, 32, 34]. Tandon et al. [5] recommended to undergo surgery between 1 and 2 weeks. In contrast, Emanuelli et al. [40] showed a significant difference for beginning of surgical treatment within 12 h after the injury. An animal-based experimental study reported damages and changes on the molecular base of the nerve within 72 h after an injury [41]. In their systematic data review, Dhaliwal et al. analyzed more than 24 articles and concluded that more than 50% of the patients benefitted from surgery regardless of timing [12]. Considering these varying recommended time frames to initiate surgical treatment, we decided to divide our patients according to their time delay from symptom onset to surgery into four categories: surgery within 1 day (3 out of 8 improved, 37%), between 1 and 3 days (1 out of 3 improved, 33%), between 3 days and 1 week (3 out of 7 improved, 42%) and more than 1 week (7 out of 12 improved, 64%). There was no significant difference in surgery delay between visual improvement groups (*p* = 0.614); however, it is necessary to additionally taken the underlying etiology into account. Therefore, we performed further subgroup analyses based on the underlying cause. Nevertheless, we also found no significant association between surgery delay and visual improvement in both trauma and tumor subgroups. These results are in concordance with the conclusion stated in the review article by Dhaliwal et al. [12]. It appears that the timing of endoscopic optic nerve decompression does not have a substantial impact on the visual outcome after the surgical procedure.

As an inflammatory blood marker, we decided to evaluate the potential influence of preoperative CRP levels on the visual outcome after endoscopic optic nerve decompression. We failed to find a significant association between preoperative CRP levels and visual outcome. Hence, higher CRP levels before surgery seem not to have negative effects on the visual outcome.

The shortcomings of our study are a small sample size, the retrospective onset and the lack of objective evaluation for visual function.

## Conclusion

Considering the success rate of vision recovery (50%) after endoscopic decompression surgery, the establishment of standard guidelines for optic nerve compressions would be an important target to improve the outcome of future patients. Regarding our results, nerve sheath incision did not result in improvement. The timing of surgery as well as elevated CRP levels did not significantly influence vision recovery. We suggest that radiological findings as indicator for surgery could help to avoid nonbeneficial surgeries. Patients with an affected nerve in preoperative imaging seem to benefit the most from a surgical intervention. In the case of an undamaged nerve, a wait-and-see strategy may be considered. The use of steroids should be re-evaluated and investigated with prospective studies analyzing their value in current therapy.
